# Integration of bioinformatics analysis and experimental validation identifies plasma exosomal miR‐103b/877‐5p/29c‐5p as diagnostic biomarkers for early lung adenocarcinoma

**DOI:** 10.1002/cam4.4788

**Published:** 2022-05-18

**Authors:** Jing Wu, Zian Feng, Rui Wang, Ang Li, Hong Wang, Xiaodong He, Zuojun Shen

**Affiliations:** ^1^ Department of Clinical Laboratory Anhui Provincial Hospital Affiliated to Anhui Medical University Hefei Anhui China; ^2^ Department of Clinical Laboratory The First Affiliated Hospital of USTC, Division of Life Sciences and Medicine, University of Science and Technology of China Hefei Anhui China; ^3^ Department of Radiation Oncology The First Affiliated Hospital of USTC, Division of Life Sciences and Medicine, University of Science and Technology of China Hefei Anhui China; ^4^ Anhui Provincial Center for Clinical Laboratories Hefei Anhui China

**Keywords:** bioinformatics, exosome, lung adenocarcinoma, miRNA, qRT‐PCR

## Abstract

The aim of this study was to identify miRNAs in plasma exosomes as noninvasive biomarkers for the early diagnosis of lung adenocarcinoma (LUAD). First, exosomal miRNA profiling of three patients with early LUAD and three patients with benign lung disease were screened by next‐generation sequencing (NGS) method. Sequencing results showed that 154 exosomal miRNAs were differentially expressed in the plasma of LUAD patients, among which 68 miRNAs were up‐regulated and 86 miRNAs were down‐regulated. GSE137140 is a GEO database containing serum miRNAs sequencing data from 1566 lung cancer patients and 1774 non‐cancer patients controls. When comparing the sequencing data, it was found that most miRNAs (37/68) up‐regulated in our LUAD group were also significantly up‐regulated in GSE137140, suggesting that circulating miRNAs in lung cancer patients may be enriched in plasma exosomes. In GSE137140, the AUC of the combination of hsa‐miR‐103b, hsa‐miR‐29c‐5p and hsa‐miR‐877‐5p was 0.873, showing great potential as new tumor markers. To our knowledge, these three exosomal miRNAs have not been reported in lung cancer research. Furthermore, bioinformatics tools were used to analyze the target genes of three candidate miRNAs, which were indeed closely related to the occurrence and development of lung cancer. Bioinformatics algorithms deduced a highly conserved sequence in the 3’‐UTR of SFRP4, FOXM1 and TMEM98 that could be bound with miR‐103b/877‐5p/29c‐5p. A luciferase assay indicated that miR‐103b/877‐5p/29c‐5p directly targeted the 3’‐UTR of SFRP4, FOXM1 and TMEM98, respectively. Finally, three candidate miRNAs were validated by qRT‐PCR in 17 early LUAD samples and 17 control plasma samples. Integration of bioinformatics analysis and experimental validation identifies, this study provides novel insights into miRNA‐related networks in LUAD. Hsa‐miR‐103b, hsa‐miR‐29c‐5p, and hsa‐miR‐877‐5p may be used as diagnostic biomarkers for early LUAD.

## Graphical abstract

Integration of bioinformatics analysis and experimental validation, this study provides novel insights into miRNA related networks in LUAD. Plasma exosomal hsa‐miR‐103b, hsa‐miR‐29c‐5p and hsa‐miR‐877‐5p may be used as diagnostic biomarkers for early LUAD.

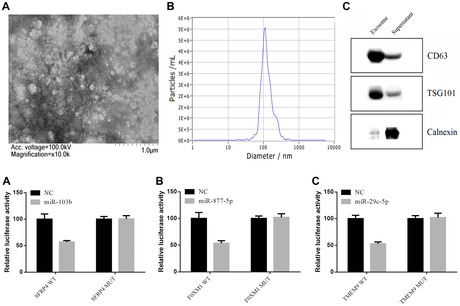

## 1 INTRODUCTION

Although morbidity and mortality rates have been declining, lung cancer remains one of the most common malignancies in both the United States and China.^1^, ^2^ Non‐small cell lung cancer (NSCLC) is the most common type of lung cancer, which can be further classified into lung adenocarcinoma (LUAD) and lung squamous cell carcinoma (LUSC). Most lung cancer patients are diagnosed as advanced, with a 5‐year survival rate of less than 15% after surgery and anticancer treatment.^3^ Currently, computed tomography (CT) has been used as a screening method for NSCLC, but its clinical application is limited by its lack of specificity and ionizing radiation.^4^ Blood‐based testing is an ideal non‐invasive diagnostic method, such as serum markers carcinoembryonic antigen (CEA) and cytokeratin 19 fragments (CYFRA21‐1) can help diagnose early lung cancer. They are generally not recommended as biomarkers for lung cancer due to their lack of sensitivity and specificity.^5^ Therefore, it is urgent to find specific biomarkers with high sensitivity to identify lung cancer.

MicroRNAs (miRNAs) are highly conserved noncoding RNAs, approximately 22 nucleotides in length, and play a central role in regulating gene expression.^6^ Currently, an increasing number of abnormally expressed miRNAs have been found in various tissues and body fluids, and have been reported as biomarkers for diagnosis, prognosis, or monitoring of NSCLC.^7^, ^8^ In human blood, miRNAs from donor cells can be incorporated into exosomes and transported through the blood to recipient cells, where they play important roles in new locations.^9^, ^10^

Exosomes are lipid bilayer vesicles released by various cells that mediate intercellular communication and are involved in the occurrence, development, and progression of cancer.^11^ Exosomes are rich in miRNAs and enhance miRNAs tolerance through lipid bilayers that protect miRNAs from RNA enzyme degradation.^12^, ^13^ Exosomal miRNAs should be the starting point for early biomarker studies, reducing the possibility of false‐negative results for miRNAs with low abundance.^12^ Several studies have explored the potential application of exosomal miRNAs in the clinical diagnosis and prognosis of lung cancer.^14^, ^15^

In this study, exosomes were isolated from plasma of patients with early LUAD and benign lung disease. Exosomal miRNA profiling of three early LUAD patients and three patients with benign lung disease was performed using NGS method to identify LUAD‐specific miRNAs. Then, bioinformatics methods were used to analyze candidate miRNAs. Luciferase assay results showed that miR‐103b / 877‐5p / 29c‐5p directly targeted the 3’‐UTR of SFRP4, FOXM1 and TMEM98, respectively. Finally, three upregulated exosomal miRNAs were confirmed by qRT‐PCR. Our work suggests that the altered expressed exosomal miRNAs in plasma may be used as a noninvasive biomarkers for the diagnosis and treatment of LUAD at an early stage.

## 2 MATERIALS AND METHODS

### 2.1 Collection of patient samples

Twenty patients with early LUAD and twenty patients with benign lung disease were included in the sequencing and validation studies. Detailed clinical data are summarized in Table S1–S2. Three early LUAD patients and three controls were randomly selected for NGS method (Table 1), and the remaining samples were further verified by qRT‐PCR. Peripheral blood samples were collected with EDTA anticoagulant tube. Peripheral blood samples were collected from all patients before treatment procedures (e.g., surgery, preoperative radiotherapy, and chemotherapy). All patients with lung cancer were confirmed to be lung adenocarcinoma by histopathology. According to the eighth edition of the American Joint Committee on Cancer (AJCC), information on TNM staging, and differentiation of the tumor was retrospectively obtained from the patient's medical records. Ethical approval was waived off.

**TABLE 1:** Clinical characteristic of the patients

	Testing set (*N* = 6)	Validation set (*N* = 34)	*p* value
Categories			
Tumor	3	17	
Control	3	17	
Gender			0.534
Male	4	17	
Female	2	17	
Age (years), median	58.83 ± 7.57	54.09 ± 15.86	0.863
TNM stage			
Ia	2	16	
Ib	1	1	

### 2.2 Isolation of plasma exosome

According to the manufacturer’s specification, ExoQuick™ (system Biosciences, CA, USA) is a commercial kit for the separation of exosomes from EDTA anticoagulated plasma. In brief, 200 μl of collected plasma was lightly mixed with 50 μl EQ reagent and placed at 4°C for 30 min. After centrifugation at 1500*g* for 30 min, the exosomes are precipitated at the bottom of the centrifuge tube.

### 2.3 Characterization and quantification of exosomes

Negative staining of exosomes suspension was performed and transmission electron microscopy was performed to identify the isolated exosomes. A total of 10 μl exosome suspension was deposited on a copper network, and phosphotungstate was coated on the grid. After drying for 2 min, 80 KV electron microscope was used for detection and imaging, and the final transmission electron microscope (HT‐7800) imaging results were obtained. Exosome suspension was treated with 200 μL RIPA lysis buffer (Beyotime, Shanghai, China) to extract protein. Electrophoresis was performed on 10% SDS‐PAGE gel, imprinted on PVDF membrane, and incubated with primary antibodies (CD63 (AB68418), TSG101 (AB125011), and calnexin (10427‐2‐AP, Promega)) at 4°C overnight. The PVDF membrane was then rinsed three times with a TBST‐containing buffer and incubated with secondary antibodies for 1.5 h at room temperature. The immune response bands were detected using a Western ECL kit (Beyotime). The exosome precipitates were resuspended with PBS and mixed evenly with a vortex agitator. Diluted samples were taken and exosome size was measured using a ZetaView device (Particle Metrix).

### 2.4 Libraries preparation and RNA‐seq analysis

High‐throughput transcriptome sequencing was carried out by the BGISEQ sequencing platform. In short, linear DNA amplification sequencing was performed using DNA nanospheres (DNB). According to the manufacturer's instructions, Unique molecular identifier (UMI) was introduced during the library construction, which was connected to the cDNA molecule in the early stage of library construction, and tagged each molecule in the original sample. RNA libraries were quantified using Agilent 2100 BioAnalyzer (Agilent Technologies). Finally, the sequencing was performed using the combined probe‐anchored polymerization (cPAS) technique according to the amplification procedure.

### 2.5 Prediction of miR‐103b/877‐5p/29c‐5p target genes

MiRWalk 2.0 (http://zmf.umm.uni‐heidelberg.de/apps/zmf/mirwalk2/miRretsys‐self.html), is an easy to access open database, which contains the prediction data obtained by machine learning algorithm.^16^ This database has a clear structure, intuitive interface, and user‐friendly design. The data in the database are free and constantly updated. The target genes of miR‐103b/877‐5p/29c‐5p were predicted using this database in conjunction with three other effective and promising target gene prediction tools for microRNAs(miRanda: http://www.microrna.org/microrna/getDownloads.do, RNA 22: https://cm.jefferson.edu/rna22v2/, Targetscan 6.2: http://www.targetscan.org/cgi‐bin/targetscan/data_download.cgi?db=vert_61).^17^, ^18^, ^19^

### 2.6 Functional enrichment analysis and transcription factor prediction

FunRich database(http://www.funrich.org/), an online annotation and analysis tool to understand bioinformatics information.^20^ The FunRich database was used to study the GO annotation of target genes and predict biological signaling pathways and transcription factors.

### 2.7 3’‐UTR luciferase assay

Bioinformatics method was used to analyze the binding of miR‐103b/877‐5p/29c‐5p to the 3’‐UTR region of SFR4, FOXM1 and TMEM98, respectively. According to the analysis results, Wild‐type (WT) and Mutant‐type (MUT) 3’‐UTR of SFRP4, FOXM1 and TMEM98 were synthesized by Hefei qinheng. Then, 293T cells were co transfected with miR‐NC and miR‐103b/877‐5p/29c‐5p mimics and WT and MUT 3’‐UTR vectors of SFRP4, FOXM1 and TMEM98 for 24 h, respectively. The luciferase activity was carried out using Luciferase Assay System (Promega, E1910).

### 2.8 Quantification of miRNA expression with qRT‐PCR

Total RNA was extracted from the plasma isolated exosomes using TRIzol reagent (Invitrogen, Carlsbad) according to the manufacturer's instructions. A similar amount of Caenorhabditis elegans cel‐miR‐39 (RiboBio) was incorporated into exosome sample for external calibration of RNA extraction, reverse transcription, and miRNA amplification. According to the manufacturer's instructions, the PrimeScriptTM RT Master Mix kit (Takara) was used to reverse transcribes the extracted total exosomal RNA to obtain cDNA. PCR amplification of cDNA was performed using TB Green® Premix Ex TaqTM II (Takara) at a total reaction volume of 20 μl. The qRT‐PCR was run on ABI QuantStudio 5 (Applied Biosystems) to amplify and detect miRNAs with TB Green dyes. The qRT‐PCR mixture was incubated at 95°C for 30 s, followed by 40 cycles of 5 s at 95°C and 60°C for 30 s. All qRT‐PCR reactions were performed in triplicates, and miRNAs expression was calculated using the comparative cycle threshold (Ct) method. Using 2^−ΔΔCt^ method compared with the foreign reference of microRNAs (cel‐miR‐39), ΔCt = Ct miRNAs ‐ Ct cel‐miR‐39, calculate the relative expression of miRNAs. Specific primers designed for miRNAs are listed in Table S2.

### 2.9 Statistical analysis

Statistical analysis was performed using Social Science Statistical Program (SPSS) 22.0 software and graphs were generated using GraphPad Prism 5.0 (GraphPad Software). The qRT‐PCR results of the LUAD group and the control group were analyzed by *t*‐test. The binary logic model is constructed by SPSS software. The diagnostic accuracy of candidate miRNAs and their combinations was evaluated by the receiver operating characteristic (ROC) curve, and the area under the curve (AUC) was calculated. *p* < 0.05 was considered statistically significant.

## 3 RESULTS

### 3.1 Characterization of isolated plasma exosomes

**FIGURE 1:**
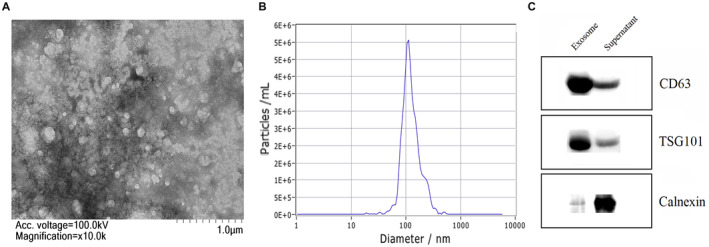
(A) TEM was used to analyze exosomes, which exhibited a cupshaped membrane morphology with a diameter of 60–150 nm. (B) The NTA results suggested that the majority of vesicle particles were mainly between 60 and 150 nm in diameter. (C) Exosomal markers CD63 and TSG101 were all detected in the exosomes enriched fractions isolated from the plasma, and Calnexin, a negative marker of exosome was absent in our isolated exosomes enriched fraction samples.

TEM, western blotting, and NTA were performed to identify the morphology and concentration of exosome particles. TEM analysis showed that the exosomes isolated were elliptical in size and ranged from 50 to 150 nm (Figure 1A). NTA results showed that the concentration of nanoparticles was 10^7^–10^11^ Particles/ml, and the particle size was 30–200 nm (Figure 1B). Western blotting showed that exosome markers CD63 and TSG101 were detected in exosomes isolated from plasma. Conversely, there was no negative marker Calnexin for exosomes (Figure 1C). The above results indicate that the plasma vesicle structure of isolated clinical patients was identified as exosomes.

### 3.2 Plasma exosomal miRNA expression profiles

**FIGURE 2:**
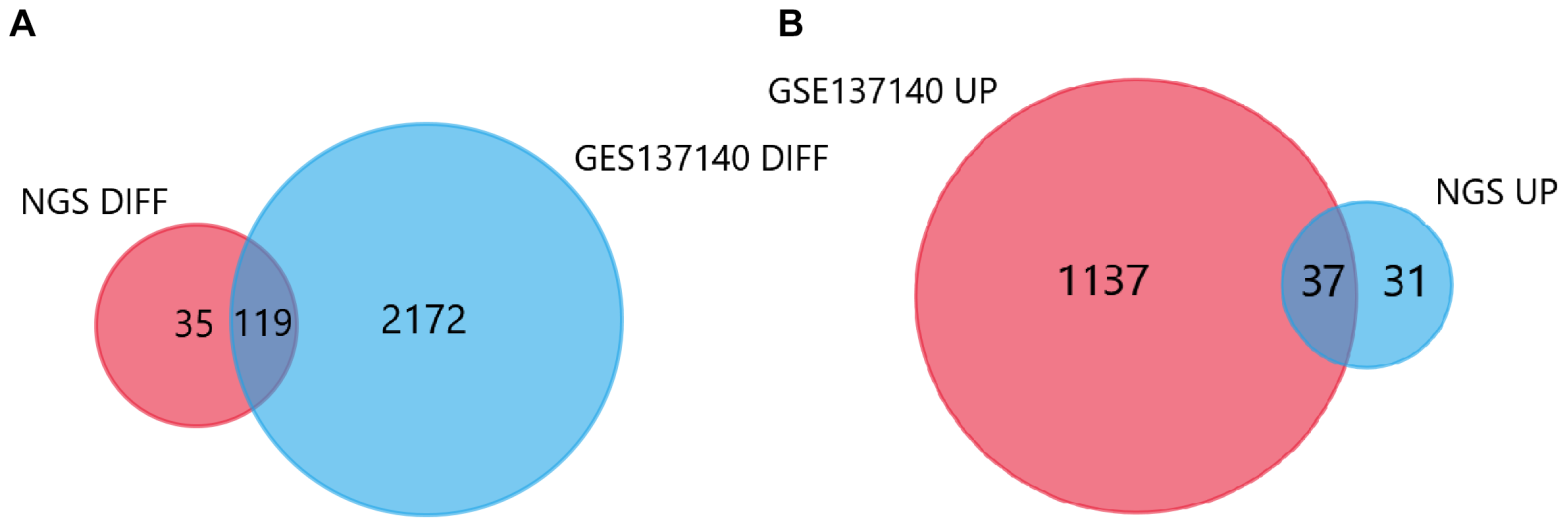
(A) A Venn diagram shows the shared differential expression of miRNAs between the TCGA dataset and our plasma exosomal miRNA dataset. (B) Upregulated miRNAs sharing differential expression between plasma exosomes and data sets.

In order to obtain the general profile of exosomal miRNA in plasma of LUAD patients, six samples (three LUAD patients and three non‐tumor controls) were tested by NGS method (bioproject ID: PRJNA699702). In the analysis of differentially expressed miRNAs (DEMs) (|log2 [FC]| > 1, *p* < 0.001), 68 miRNAs were upregulated, and 86 were downregulated in LUAD patients. When analyzing disorder miRNAs of LUAD, Venn diagram was used to identify overlapped and nonoverlapped miRNAs. Compared with the differentially expressed miRNAs in the GEO database, 119 miRNAs were differentially expressed in these two analyses (77.3%) (Figure 2A). Among the 68 upregulated miRNAs, 37 overlapped with the database (Figure 2B). Researchers have studied some miRNAs with significant differences in sequencing data. For example, hsa‐miR‐200a‐3p is upregulated in peripheral blood and exosomes of LUAD patients, and its expression level is significantly higher than that of patients with tubercular pulmonary disease and other benign pulmonary lesions.^21^ Compared with the control group, the plasma and exosomal miR‐660‐5p of NSCLC patients were significantly increased, and miR‐660‐5p may inhibit the proliferation, migration, and invasion of NSCLC by targeting KLF9, providing a potential therapeutic target for NSCLC.^15^

### 3.3 Validation of the miRNA‐seq data

**FIGURE 3:**
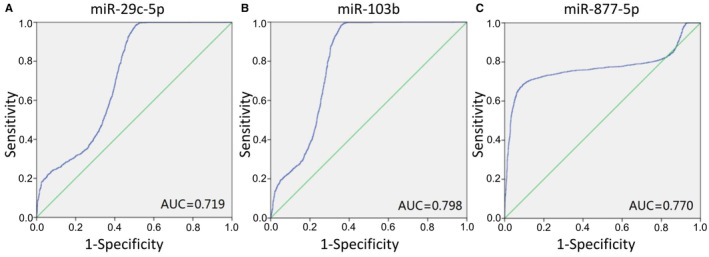
Validation of miRNA as a biomarker for lung cancer. (A) miR‐29c‐5p. (B) miR‐103b. (C) miR‐877‐5p. The ROC curve of the microarray of GSE137140 is shown in blue.

Among all the differentially expressed candidate exosomal miRNAs, three new miRNAs with high expression in lung adenocarcinoma were selected for validation. So far, the miRNAs have not been reported in the literature after a PubMed search. In GSE137140, hsa‐miR‐103b, hsa‐miR‐29c‐5p, and hsa‐miR‐877‐5p achieved an AUC of 0.798(95% confidence interval [CI]: 0.784–0.813), 0.719 (95% CI: 0.703–0.736), and 0.770 (95% CI: 0.752–0.788) (Figure 3), respectively. In brief, all three miRNAs provided meaningful AUC values, which were of great significance in distinguishing patients with lung cancer from the control group.

**FIGURE 4:**
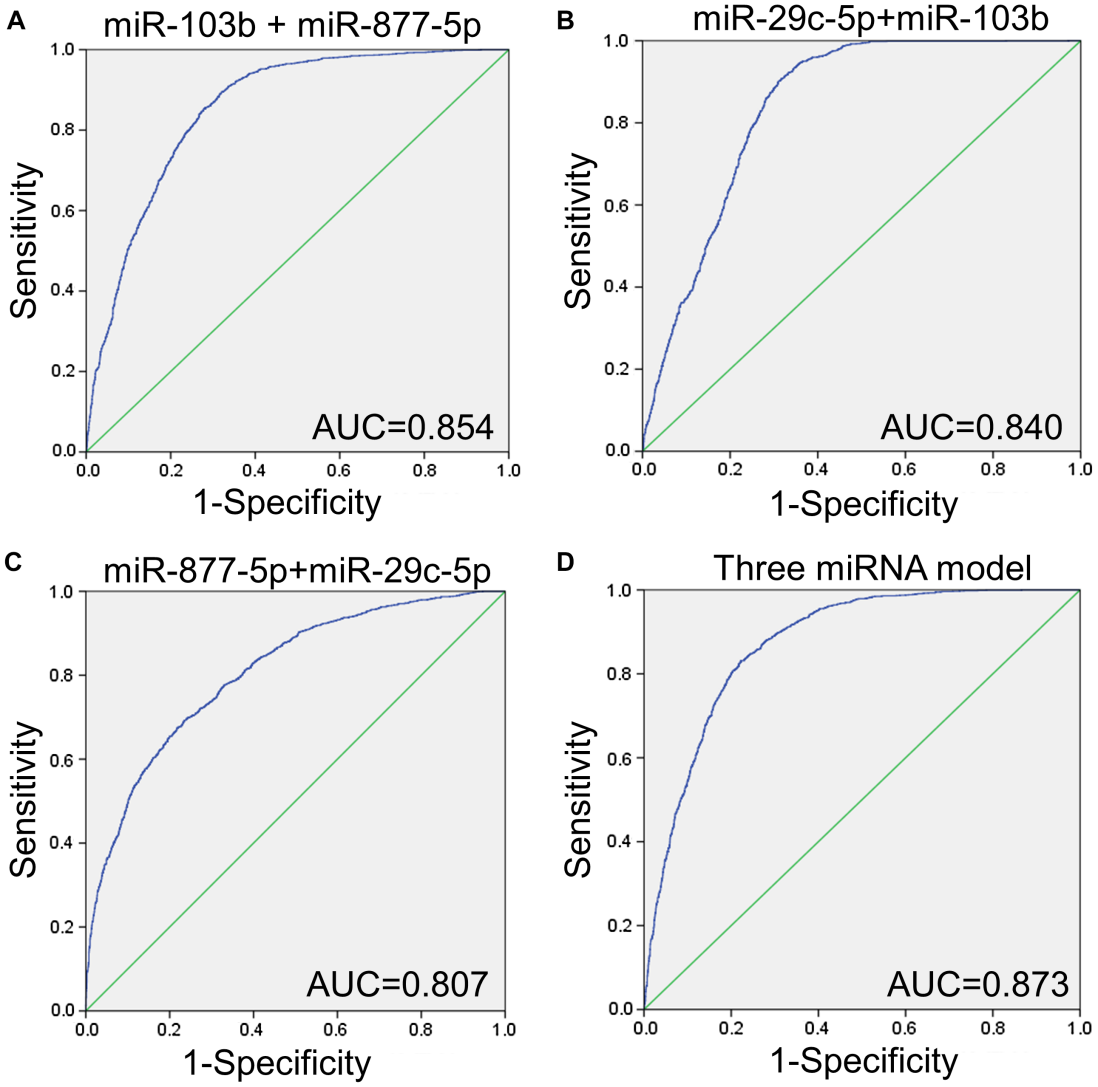
MiRNA panel validates the performance of biomarkers for lung cancer. (A) hsa‐miR‐877‐5p + hsa‐miR‐103b. (B) hsa‐miR‐29c‐5p + hsa‐miR‐103b. (C) hsa‐miR‐877‐5p + hsa‐miR‐29c‐5p. (D) hsa‐miR‐103b + hsa‐miR‐29c‐5p + hsa‐miR‐877‐5p. The ROC curve of the microarray of GSE137140 is shown in blue.

Furthermore, the integration of the two candidate miRNAs was verified by using a logical model, and better performance was obtained than that of a single miRNA. The formula for the logical models are as follows: hsa‐miR‐103b + hsa‐miR‐877‐5p = hsa‐miR ‐ 103b * 0.488 + hsa‐miR‐877‐5p * 0.459 ‐ 1.627, hsa‐miR‐29c‐5p + hsa‐miR‐103b = hsa‐miR‐103b * 0.445 + hsa‐miR‐29c‐5p * 0.314 ‐ 0.109, hsa‐miR‐877‐5p + hsa‐miR‐29c‐5p = hsa‐miR‐877‐5p * 0.458 + hsa‐miR‐29c‐5p * 0.363 ‐ 1.892, hsa‐miR‐103b + hsa‐miR‐29c‐5p + hsa‐miR‐877‐5p = hsa‐miR‐103b * 0.432 + hsa‐miR‐877‐5p * 0.423 + hsa‐miR‐29c‐5p * 0.28 ‐ 1.549. It is worth noting that hsa‐miR‐103b plus hsa‐miR‐877‐5p exhibited an AUC of 0.854 (95% CI: 0.842‐0.866) (Figure 4A). The AUC of hsa‐miR‐29c‐5p plus hsa‐miR‐103b and hsa‐miR‐877‐5p plus hsa‐miR‐29c‐5p is 0.840 (95% CI: 0.828‐0.853) and 0.807 (95% CI: 0.794‐0.821) respectively (Figure 4B, and 4C). After the integration of hsa‐miR‐103b plus hsa‐miR‐29c‐5p plus hsa‐miR‐877‐5p, the AUC value was increased to 0.873 (95% CI: 0.862‐0.884) (Figure 4D).

### 3.4 Bioinformatics analysis of miR‐103b/877‐5p/29c‐5p target genes

For hsa‐miR‐103b, hsa‐miR‐877‐5p, and hsa‐miR‐29c‐5p, the four tools found 280, 650, and 26 target genes, respectively (Figure S1). In order to better understand the functions and regulatory patterns of hsa‐miR‐103b, hsa‐miR‐877‐5p, and hsa‐miR‐29c‐5p at the cellular level, 280, 650, and 26 target genes of hsa‐miR‐103b, hsa‐miR‐877‐5p, and hsa‐miR‐29c‐5p were annotated by network analysis and the enrichment of biological signal pathway was analyzed. Regarding cellular components (CC), target genes of hsa‐miR‐103b were usually enriched in cytoplasm, plasma membrane, BRCA1‐a complex and myosin complex (Figure S2A). The results show that terms such as signal transduction, transcription, reproduction and cell‐cell signaling were enriched in GO biological process (BP) (Figure S2B). Regarding GO molecular function (MF), these 280 targets were mainly enriched in protein serine/threonine kinase activity and GTP binding. (Figure S2C). Furthermore, C‐MYC transcriptional activation and transport of organic anions were both enriched by biological pathway enrichment (Figure S2D). Terms such as cytoplasm, plasma membrane, and Golgi apparatus were enriched in GO CC (Figure S3A). The results of hsa‐miR‐877‐5p showed that GO BP was enriched in terms of cell communication and signal transduction (Figure S3B). Regarding GO MF, terms such as Auxiliary transport activity, ATPase activity, and phosphoric diester hydrolase were significantly enriched (Figure S3C). In the biological pathway enrichment analysis, terms such as JNK signaling pathway and integrins in angiogenesis and other pathways were enriched (Figure S3D). Regarding GO CC, target genes of hsa‐miR‐29c‐5p were usually enriched in endosome, early endosome, node of Ranvier and protein transporting two sector ATPase complex (Figure S4A). The results show that terms such as cell communication and signal transduction were enriched in GO BP (Figure S4B). Regarding GO MF, these 26 targets were mainly enriched in transporter activity, transmembrane receptor tyrosine kinase activity, and T‐cell receptor activity (Figure S4C). Transmission across chemical synapses, adenylate cyclase activating pathway and rapid glucocorticoid signaling pathways were enriched by biological pathway enrichment (Figure S4D).

**FIGURE 5:**
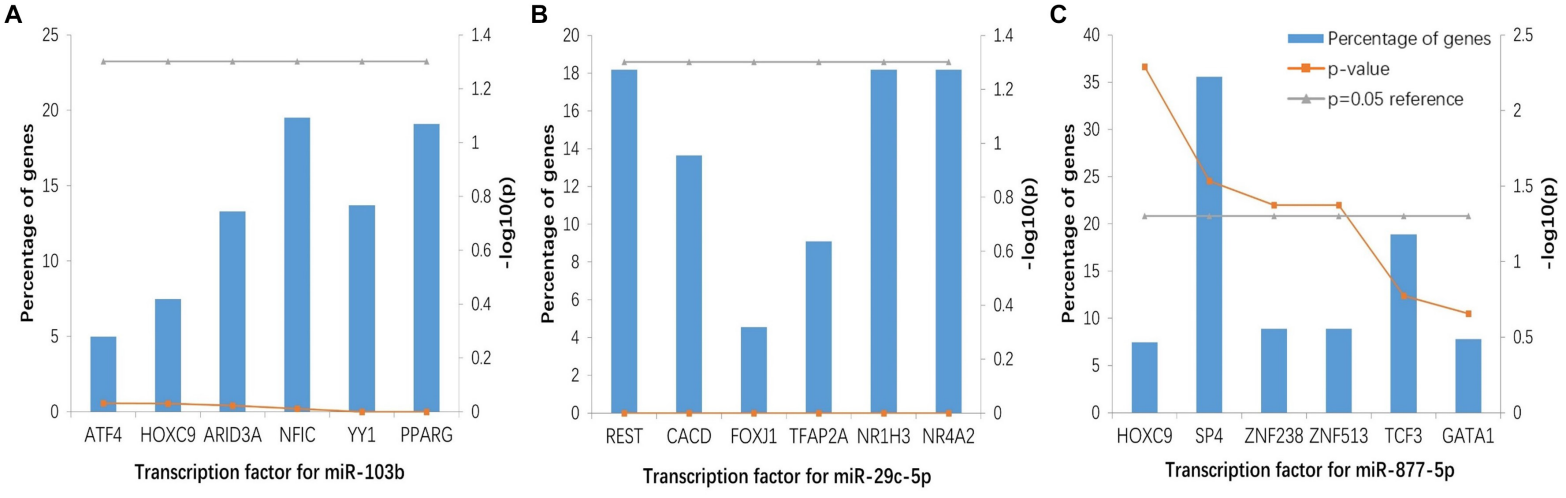
Enriched transcription factors by miRNA target genes. (A) The transcription factor for hsa‐miR‐103b target genes. (B) The transcription factor for hsa‐miR‐29c‐5p target genes. (C) The transcription factor for hsa‐miR‐877‐5p target genes.

The enriched transcription factors for the target genes of hsa‐miR‐103b were NFIC, PPARG, YY1, ARIDA, and HOXC9 (Figure 5A). The transcription factors of hsa‐miR‐29c‐5p target genes mainly include REST, NR1H3, NR4A2, CACD and TFAP2A (Figure 5B). SP4, TCF3, ZNF513, ZNF238 and HOXC9 were mainly enriched in hsa‐miR‐877‐5p target genes (Figure 5C).

### 3.5 MiR‐103b/877‐5p/29c‐5p directly targets the SFRP4, FOXM1 and TMEM98 3’‐UTR

**FIGURE 6:**
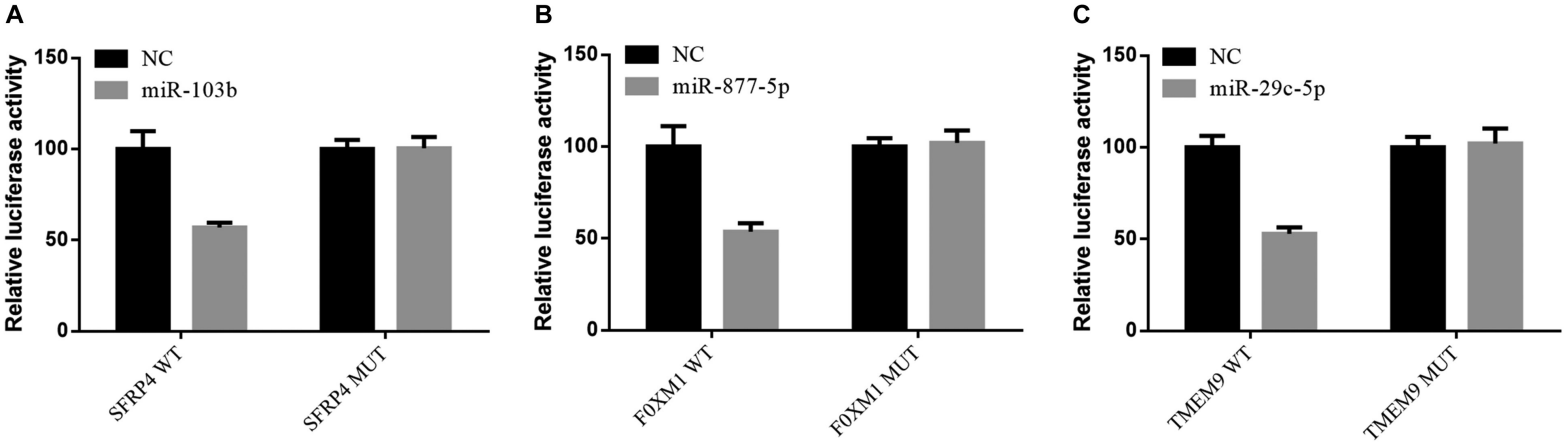
(A) Decrease activity of dual luciferase assay clarified that miR‐103b bind to the 3’‐UTR of SFRP4 in 239T cells while the mutant vector showed no changes. (B) Decrease activity of dual luciferase assay clarified that miR‐877‐5p bind to the 3’‐UTR of FOXM1 in 239T cells while the mutant vector showed no changes. (C) Decrease activity of dual luciferase assay clarified that miR‐29c‐5p bind to the 3’‐UTR of TMEM9 in 239T cells while the mutant vector showed no changes.

In order to investigate possible miRNA‐target gene interactions, we used various bioinformatics based methods described above to predict the target of miR‐103b/877‐5p/29c‐5p. In this study, the predicted binding sites of miR‐103b in SFRP4 mRNA 3’‐UTR, miR‐877‐5p in FOXM1 mRNA 3’‐UTR and miR‐29c‐5p in TMEM98 mRNA 3’‐UTR were selected (Figure S5). Subsequently, in order to evaluate whether miR‐103b/877‐5p/29c‐5p directly regulates the expression of SFRP4, FOXM1 and TMEM98, 3’‐UTRs carrying SFRP4, FOXM1 and TMEM98 mRNA and luciferase reporter plasmids (wild type and mutant) carrying miR‐103b/877‐5p/29c‐5p binding sites or mutant binding sites were cotransfected into 293T cells with miR‐103b/877‐5p/29c‐5p mimics and miRNA control, respectively Luciferase reporter gene analysis showed that 293T cells co‐transfected with miR‐103b/877‐5p/29c‐5p mimics and plasmids of SFRP4, FOXM1 and TMEM98‐WT‐3’‐UTR (wild‐type and mutant) showed significantly lower luciferase activity in wild‐type cells compared with mutant ones. These results indicated that SFRP4, FOXM1 and TMEM98 were the direct targets of miR‐103b/877‐5p/29c‐5p, respectively (Figure 6).

### 3.6 Expression of Exosomal Mir‐103b/877‐5p/29C‐5p

**FIGURE 7:**
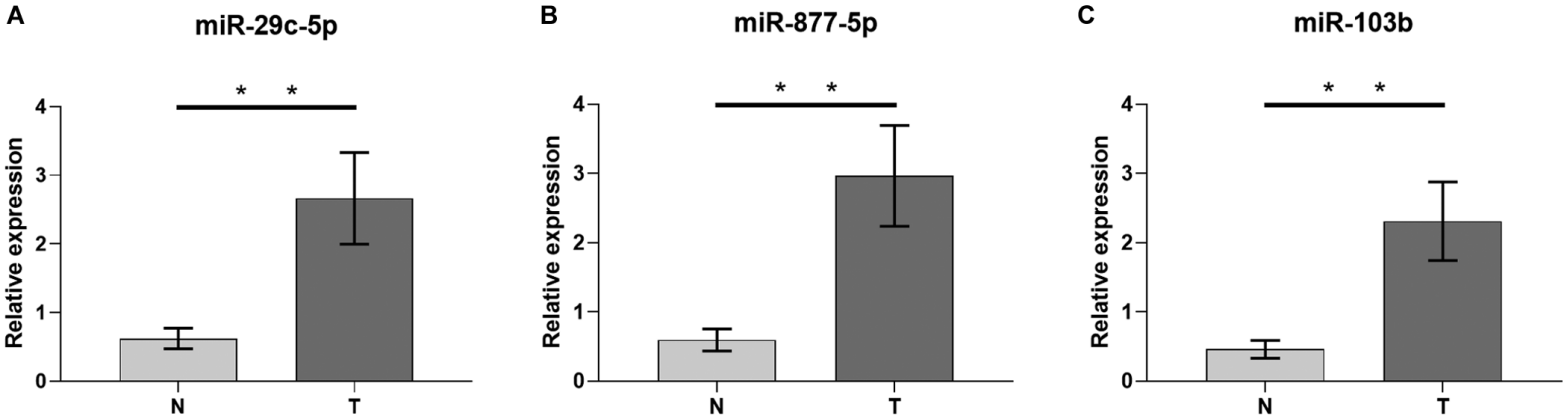
Expression levels of the three miRNAs in the plasma exosome of 17 LUAD patients and 17 non‐tumor patients. (A) miR‐29c‐5p; (B) miR‐877‐5p; (C) miR‐103b. N, non‐tumor patients; T, tumor patients. **p < 0.05.

The technology of qRT‐PCR was applied in the validation set to study the robustness of the sequencing data, including 17 additional LUAD plasma exosomes samples and 17 control samples. ΔCt was evaluated for the three miRNAs normalized to cel‐miR‐39. As shown in Figure 7, hsa‐miR‐103b, hsa‐miR‐877‐5p and hsa‐miR‐29c‐5p in plasma exosomes of LUAD patients were significantly up‐regulated compared with the control group (*p* < 0.05), which was consistent with the previous sequencing results. qRT‐PCR data validated the robustness of sequencing data analysis.

## 4 DISCUSSION

NSCLC accounts for about 85% of all lung cancers, and LUAD is one of the main histological subtypes.^22^ MiRNAs are involved in the occurrence and development of a variety of malignant tumors, and may be a treasure house for cancer diagnosis and treatment, including lung cancer.^23^ Further study of exosomal miRNAs may help to understand their value in lung cancer. To our knowledge, this study was the first to report that hsa‐miR‐29c‐5p, hsa‐miR‐103b, and hsa‐miR‐877‐5p were enriched in plasma exosomes from patients with early lung adenocarcinoma.

Tumor cells exchange basic information between local and distant recipient cells by releasing exosomes that carry proteins, lipids, and nucleic acids. Exosomes can be used as prospective noninvasive biomarkers of lung cancer, which are widely distributed in body fluids.^24^ The lipid membrane structure of exosomes protects their contents from degradation by ribonuclease.^25^ This enhances the potential of exosomal miRNAs as biomarkers for cancer diagnosis and treatment.^26^, ^27^

The AUC values of hsa‐miR‐103b, hsa‐miR‐29c‐5p, and hsa‐miR‐877‐5p in the GSE137140 were verified to be 0.798, 0.719, and 0.770, respectively. Many other studies have suggested that circulating miRNAs can be used as biomarkers for lung cancer, and the AUC of most studies is about 0.75.^28^, ^29^ The effect of multiple miRNAs integration is better than that of single miRNA in differentiating early lung cancer patients.^30^, ^31^ Here, the AUC obtained by combining only two miRNAs (hsa‐miR‐877‐5p and hsa‐miR‐29c‐5p) was 0.807, while the combined AUC of three biomarkers (hsa‐miR‐103b, hsa‐miR‐29c‐5p and hsa‐miR‐877‐5p) was 0.873. There is evidence that hsa‐miR‐29c‐5p is an important regulator of CDC42 and DNMT3A genes in the pathogenesis of gastric cancer.^32^ Yu et al. showed that hsa‐miR‐877‐5p can play a role of self‐protection or adaptation in liver injury induced by acetaminophen.^33^ In addition, most miRNAs are tissue specific and capable of reflecting pathological characteristics. In addition, most miRNAs are tissue specific and capable of reflecting pathological features. For example, plasma exosomal miRNAs (let‐7b‐3p, miR‐139‐3p, miR‐150‐3p, miR‐145‐3p) can distinguish patients with early colorectal cancer from healthy controls.^34^ Lai et al. believed that exosomal miRNAs have a better diagnostic value for pancreatic cancer.^35^ It is necessary to further study these exosomal miRNAs in the plasma of a large number of LUAD patients to prove their potential as non‐invasive biomarkers of LUAD.

More and more studies have found that miRNAs play a key role in tumor angiogenesis, proliferation, metabolism, migration, and invasion.^36^ So far, the function of most miRNAs has been unknown. With the development of high‐throughput miRNA detection techniques, more and more new miRNAs have been discovered. MiRNA has been widely studied in the occurrence and development of lung cancer.^37^ Although the relationship between hsa‐miR‐29c‐5p, hsa‐miR‐103b, and hsa‐miR‐877‐5p in lung cancer has not been reported, the results suggest that they may play an important role in the pathogenesis of LUAD.

Four promising miRNA target prediction tools were used, such as miRwalk 2.0,^16^ RNA 22,^18^ miRanda,^17^ and Targetscan 6.2^19^ to explore the target genes of hsa‐miR‐103b, hsa‐miR‐877‐5p, and hsa‐miR‐29c‐5p. Target genes of 280, 650, and 26 were found in the predicted cross points of the four tools, indicating that they may be promising target genes of hsa‐miR‐103b, hsa‐miR‐877‐5p, and hsa‐miR‐29c‐5p. Some of these target genes, such as IL7R,^38^ DDR1,^39^ FZD3,^40^ BCL9,^41^ RAD518^42^ etc., have been confirmed to be involved in the occurrence and development of lung cancer in previous studies, which also proved that miRNAs may play an important role in the occurrence and development of lung cancer. Go annotation and biosignaling pathway analysis were performed using 280, 650, and 26 target genes to reveal the main regulatory patterns of hsa‐miR‐103b, hsa‐miR‐877‐5p, and hsa‐miR‐29C‐5p. The results suggest a potential association between miRNAs and lung cancer. The down‐regulation of NFIC expression may be a key transcription factor in the development of LUSC by regulating genes involved in cell cycle and DNA replication.^43^ HOXC9 promotes the invasion and proliferation of NSCLC cells, and miR‐495 binds to HOXC9 3’‐UTR region to inhibit its expression. Hsa‐circ‐0020123 may play an antitumor role in NSCLC by regulating miR‐495/HOXC9 axis.^44^ Many studies have shown that PPAR gamma activation can hinder the progress of lung cancer, and PPARG ligand can be used as a potential therapeutic agent for lung adenocarcinoma and PPARG can also effectively treat lung squamous cell carcinoma.^45^ Liu's data shows that TFAP2A induced the high expression of KRT16 in LUAD, and KRT16 promoted the tumorigenicity of LUAD through EMT migration, invasion, and proliferation.^46^ Huang’s study showed that YY1 was up‐regulated in lung cancer tissues and affected cell proliferation, migration and invasion, and suggested that YY1 played a key role in the progression of lung cancer partly through the activation of its downstream target PVT1.^47^

In addition, the robustness of miRNA‐Seq data was verified by qRT‐PCR analysis of three candidate miRNAs in the cohort. Therefore, the results indicate that hsa‐miR‐103b, hsa‐miR‐29c‐5p, and hsa‐miR‐877‐5p have great potential as markers of lung cancer. This provides a new way of thinking for finding new potential diagnostic biomarkers and also provides a direction for studying the mechanism of action of these miRNAs in LUAD. However, based on the potential interaction of target gene, more participants are required to participate in whether these miRNAs play a role in the occurrence and development of LUAD.

## 5 CONCLUSIONS

In conclusion, next‐generation sequencing and bioinformatics methods were used to screen plasma exosomal miRNAs closely related to LUAD. Our work first provided a preliminary image of the differential expression of plasma and foreign secrete miRNAs in LUAD patients Further, dual luciferase assay confirmed that downstream target genes SFRP4, FOXM1 and TMEM98 may directly bind to miR‐103b, miR‐877‐5p and miR‐29c‐5p, respectively. Compared with the control group, the expression level of miR‐103b/877‐5p/ 29C‐5p was upregulated in LUAD patients, suggesting that these three plasma exosomal miRNAs may be more specific, sensitive, and stable noninvasive diagnostic biomarkers in early LUAD patients.

## AUTHOR CONTRIBUTIONS

JW and ZF designed and performed the research; RW, AL, HW, XH, and ZS analyzed the data; JW wrote the manuscript. All authors read and approved the final manuscript.

## CONFLICT OF INTEREST

The authors declare that there is no conflict of interest.

## ETHICS APPROVAL AND CONSENT TO PARTICIPATE

Ethical approval was waived off. ID number: 2021‐RE‐012.

## Supporting information


Figure S1

Figure S2

Figure S3

Figure S4

Figure S5

Table S1–S2

## Data Availability

The datasets used and/or analyzed during the current study are available from the corresponding author upon reasonable request.
