# Supplementary figures and images for: Integration of bioinformatics analysis and experimental validation identifies plasma exosomal miR‐103b/877‐5p/29c‐5p as diagnostic biomarkers for early lung adenocarcinoma
Source: Cancer Med. 2022 May 18;11(23):4411–21. doi: 10.1002/cam4.4788 (PMC9741994; doi:10.1002/cam4.4788)

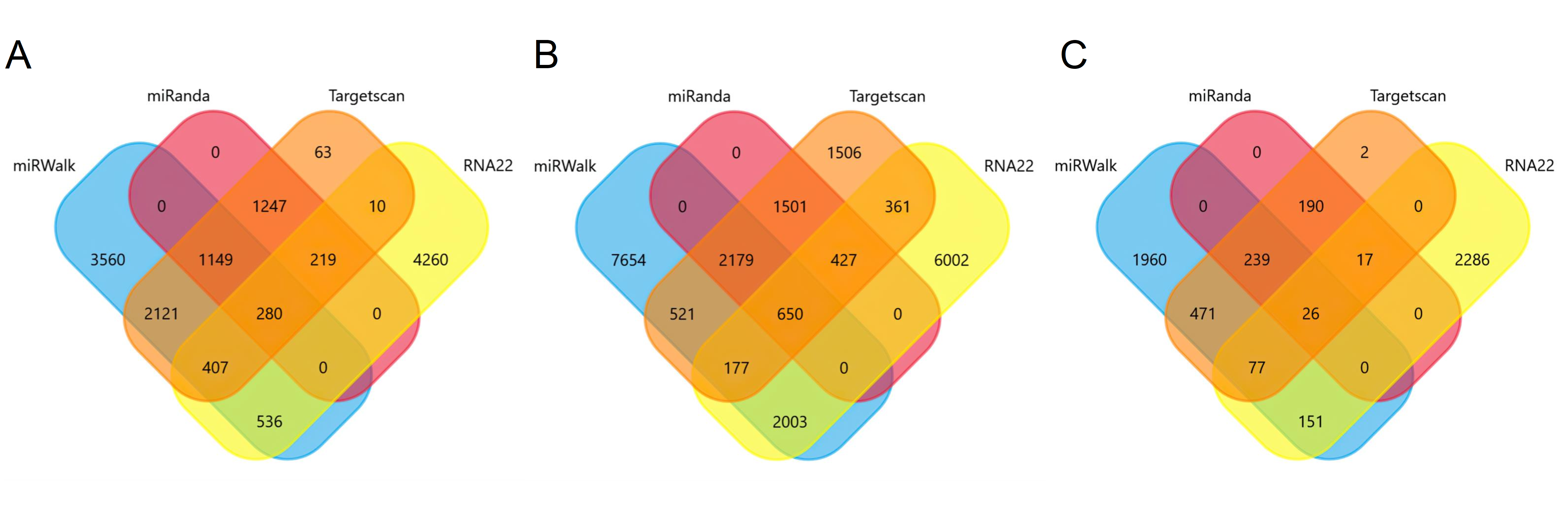

Supplement: Supplementary file 1 — Figure S1 [file CAM4-11-4411-s001.tiff]

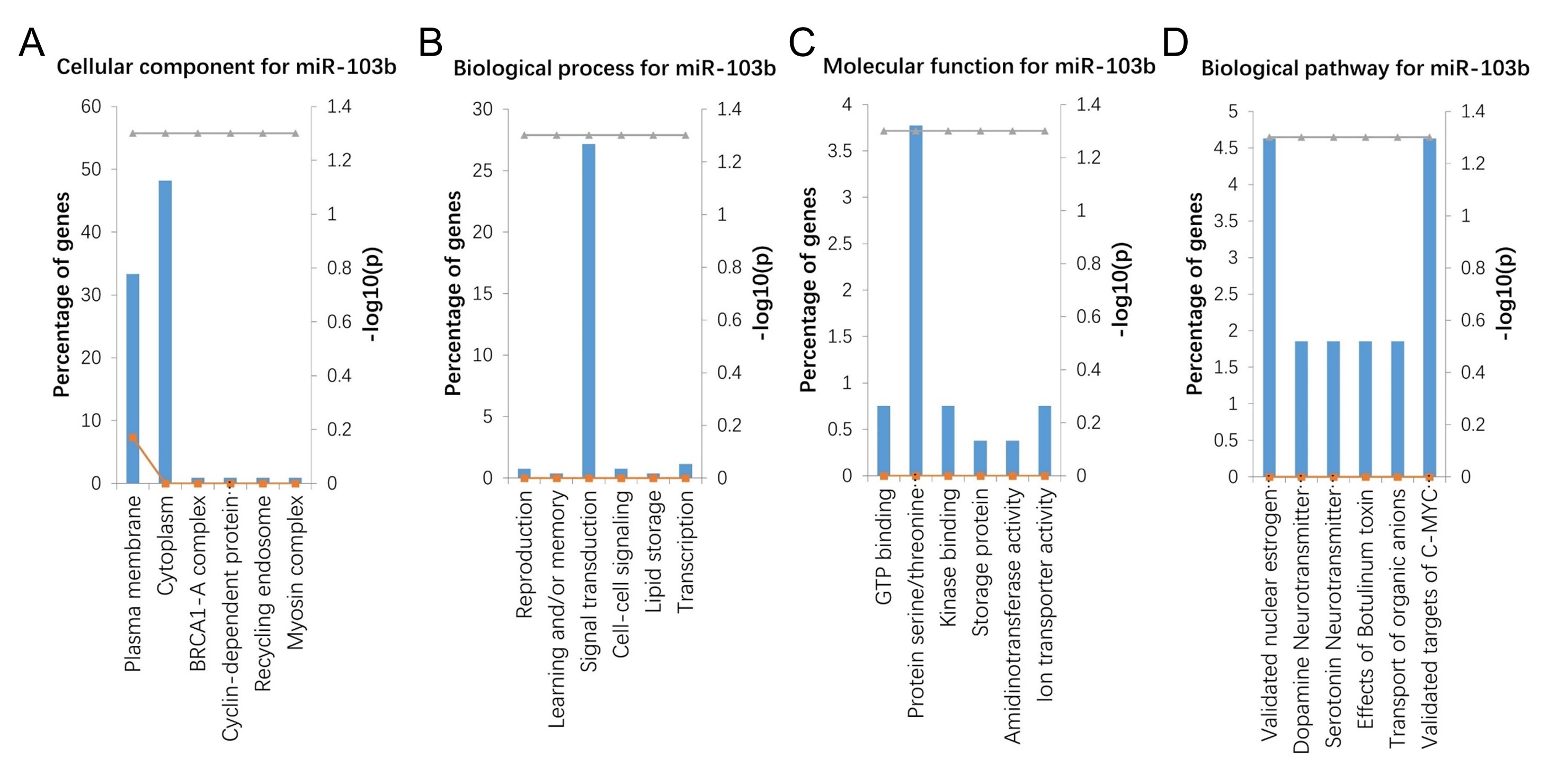

Supplement: Supplementary file 2 — Figure S2 [file CAM4-11-4411-s003.tiff]

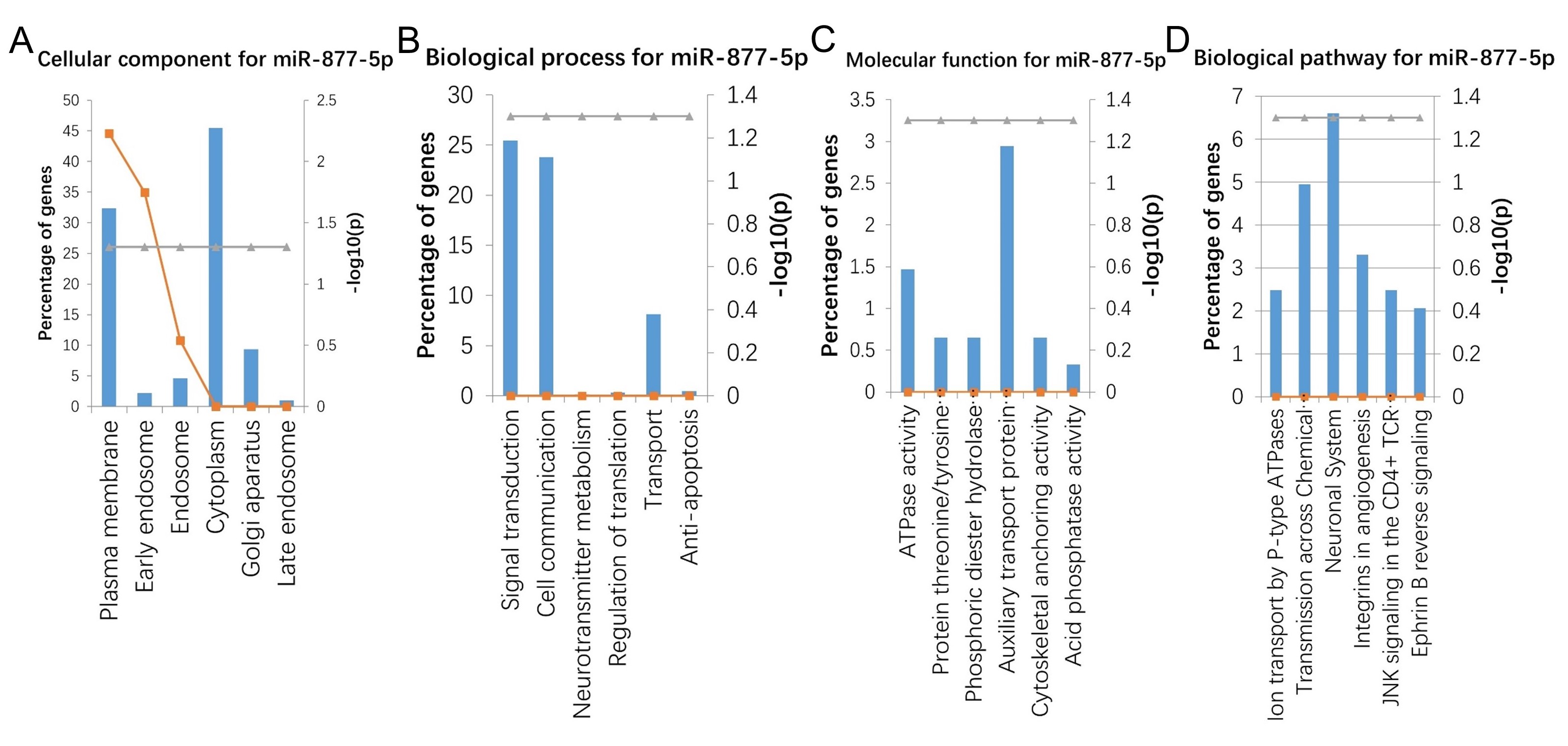

Supplement: Supplementary file 3 — Figure S3 [file CAM4-11-4411-s006.tiff]

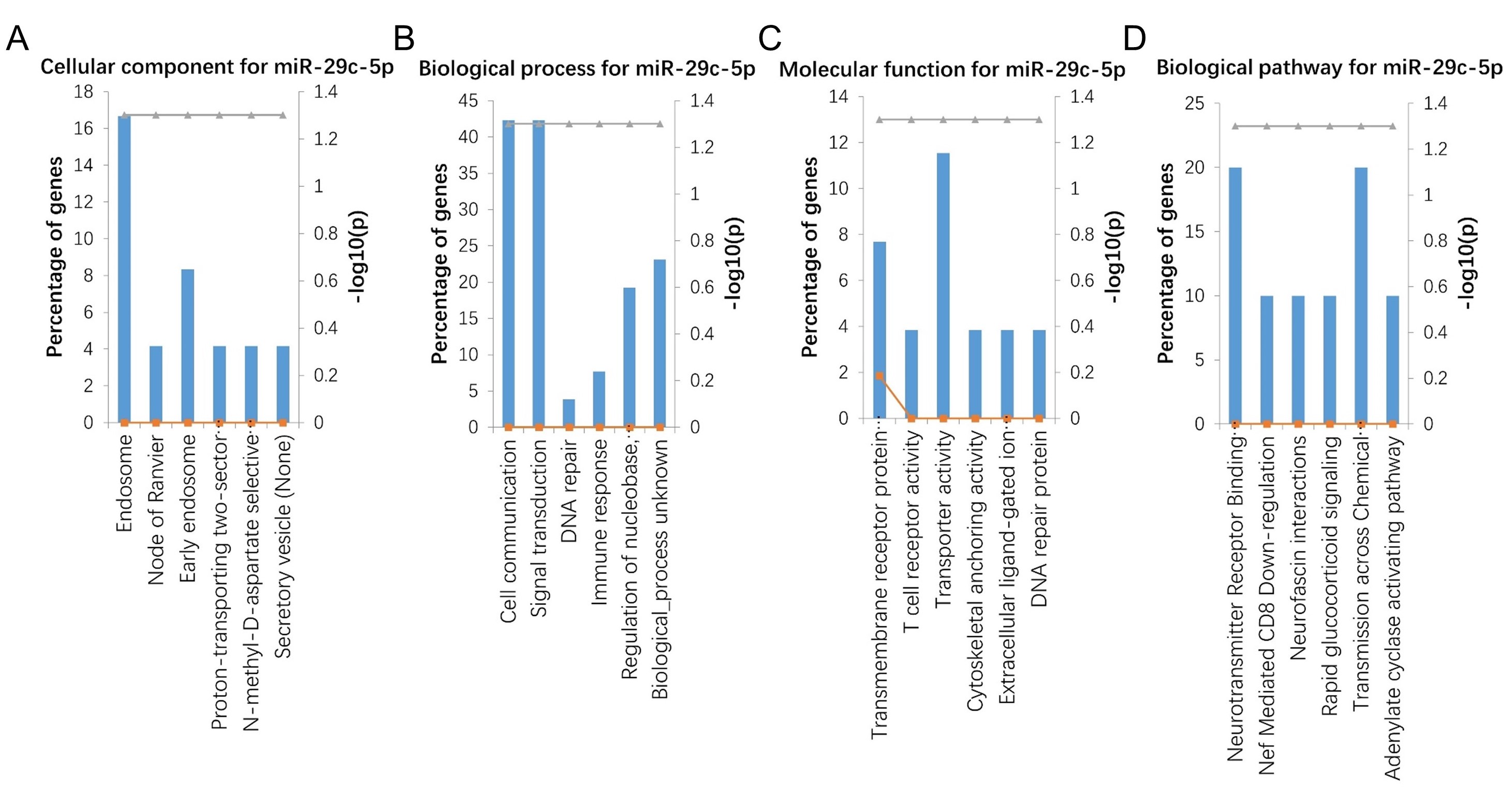

Supplement: Supplementary file 4 — Figure S4 [file CAM4-11-4411-s004.tiff]

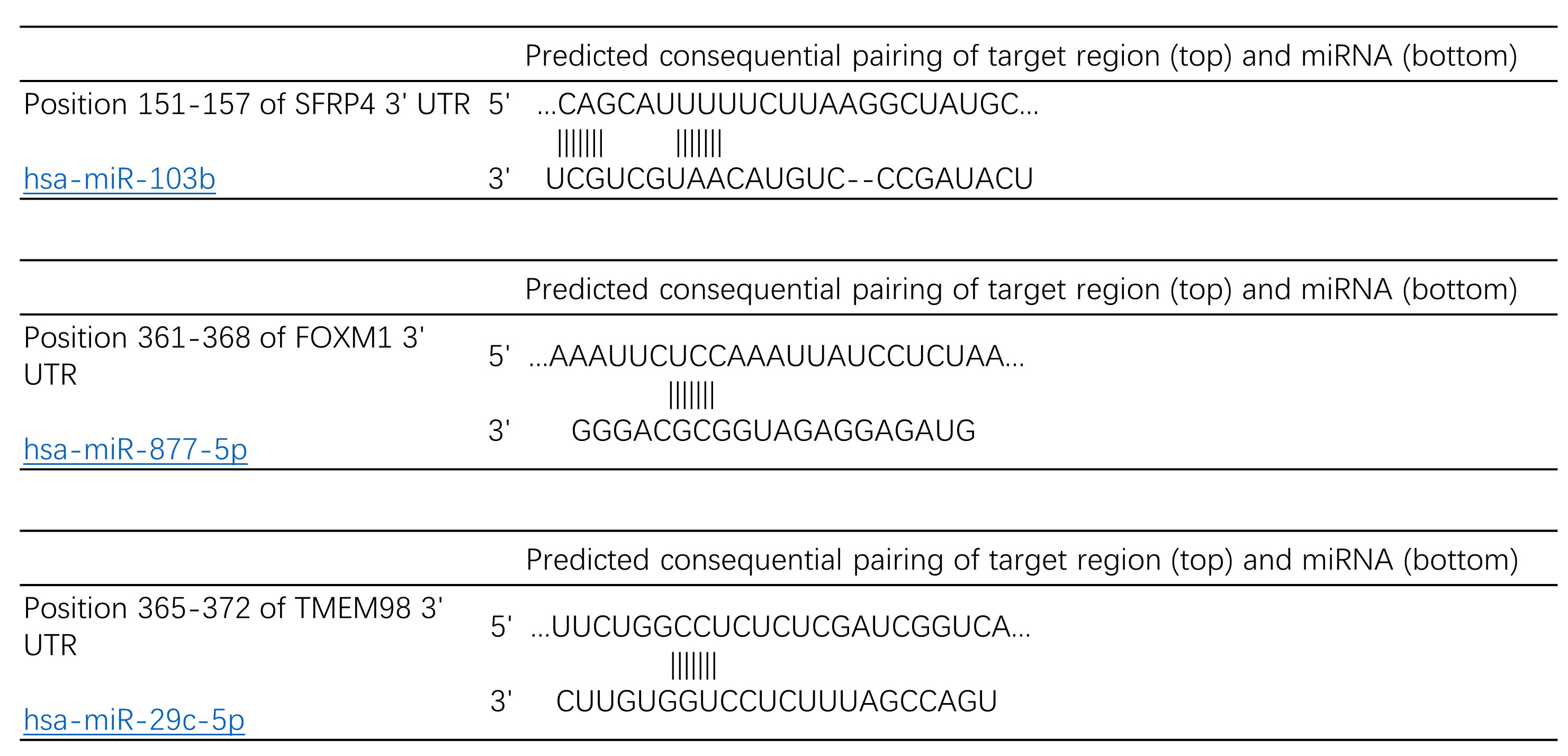

Supplement: Supplementary file 5 — Figure S5 [file CAM4-11-4411-s005.tiff]
