# Supplementary material for: Integration of bioinformatics analysis and experimental validation identifies plasma exosomal miR‐103b/877‐5p/29c‐5p as diagnostic biomarkers for early lung adenocarcinoma
Source: Cancer Med. 2022 May 18;11(23):4411–21. doi: 10.1002/cam4.4788 (PMC9741994; doi:10.1002/cam4.4788)
Supplement: Supplementary file 6 — Table S1–S2 [file CAM4-11-4411-s002.doc]

Table S1. Information of 40 patients

| Patients number | Age | Gender | TNM stage／Disease |
| --- | --- | --- | --- |
| Case 1 | 64 | Female | TbN0M0 (IA) |
| Case 2 | 70 | Male | T2aN0M0 (IB) |
| Case 3 | 60 | Male | T1bN0M0 (IA) |
| Case 4 | 40 | Male | TbN0M0 (IA) |
| Case 5 | 31 | Female | T1aN0M0 (IA) |
| Case 6 | 45 | Female | T1aN0M0 (IA) |
| Case 7 | 48 | Female | T1cN0M0 (IA) |
| Case 8 | 52 | Female | T1aN0M0 (IA) |
| Case 9 | 43 | Female | T1aN0M0 (IA) |
| Case 10 | 75 | Female | T2bN0M0 (IB) |
| Case 11 | 72 | Female | T1cN0M0 (IA) |
| Case 12 | 75 | Male | T1cN0M0 (IA) |
| Case 13 | 86 | Male | T1bN0M0 (IA) |
| Case 14 | 64 | Male | TisN0M0 (IA) |
| Case 15 | 56 | Female | T1aN0M0 (IA) |
| Case 16 | 35 | Male | T1aN0M0 (IA) |
| Case 17 | 50 | Female | T1aN0M0 (IA) |
| Case 18 | 49 | Male | T1aN0M0 (IA) |
| Case 19 | 56 | Female | T1aN0M0 (IA) |
| Case 20  Case 21  Case 22  Case 23  Case 24  Case 25  Case 26  Case 27  Case 28  Case 29  Case 30  Case 31  Case 32  Case 30  Case 34  Case 35  Case 36  Case 37  Case 38  Case 39  Case 40 | 47  57  49  53  35  70  20  42  58  71  79  71  74  51  35  64  36  43  63  52  51 | Female  Female  Male  Male  Male  Male  Female  Female  Male  Male  Male  Female  Male  Male  Female  Male  Male  Female  Male  Female  Male | T1aN0M0 (IA)  Pneumonic pseudotumor  Pulmonary granulomas  Pulmonary granulomas  Pneumonic nodule  Pneumonic pseudotumor  Lung pneumothorax  Pulmonary bulla  Pulmonary chondrohamartoma  Organized pneumonia  Lung granulomatous  Pneumonic mass  Benign pulmonary disease  Pulmonary granulomas  Pneumonic pseudotumor  Pneumonic mass  Pulmonary granulomas  Pneumonic pseudotumor  Pneumonic mass  Pneumonic pseudotumor  Benign lung tumor |

Table S2. Primers of miRNA sequences

| miRNA ID | Primer sequence 5’-3' |
| --- | --- |
| hsa-miR-103b | TCATAGCCCTGTACAATGCTGCT |
|  | ATCCAGTGCAGGGTCCGAGG |
| hsa-miR-877-5p | GTAGAGGAGATGGCGCAGGG |
|  | ATCCAGTGCAGGGTCCGAGG |
| hsa-miR-29c-5p | TGACCGATTTCTCCTGGTGTTC |
|  | ATCCAGTGCAGGGTCCGAGG |
